# From Threat to Relief: Expressing Prejudice toward Atheists as a Self-Regulatory Strategy Protecting the Religious Orthodox from Threat

**DOI:** 10.3389/fpsyg.2017.00873

**Published:** 2017-05-29

**Authors:** Małgorzata Kossowska, Paulina Szwed, Aneta Czernatowicz-Kukuczka, Maciek Sekerdej, Miroslaw Wyczesany

**Affiliations:** Institute of Psychology, Jagiellonian UniversityKraków, Poland

**Keywords:** religious orthodoxy, threat from value-violating groups, cardiovascular reactivity, prejudice

## Abstract

We claim that religious orthodoxy is related to prejudice toward groups that violate important values, i.e., atheists. Moreover, we suggest that expressing prejudice may efficiently reduce the threat posed by this particular group among people who hold high levels, but not low levels, of orthodox belief. We tested these assumptions in an experimental study in which, after being exposed to atheistic worldviews (value-threat manipulation), high and low orthodox participants were allowed (experimental condition) or not (control condition) to express prejudice toward atheists. Threat was operationalized by cardiovascular reactivity, i.e., heart rate (HR); the higher the HR index, the higher the threat. The results found that people who hold high (vs. low) levels of orthodox belief responded with increased HR after the threat manipulation. However, we observed decreased HR after the expression of prejudice toward atheists among highly orthodox participants compared to the control condition. We did not find this effect among people holding low levels of orthodox belief. Thus, we conclude that expressing prejudice toward this particular group may be an efficient strategy to cope with the threat posed by this group for highly orthodox people. The results are discussed in light of previous findings on religious beliefs and the self-regulatory function of prejudice.

## Introduction

Large-scale uncertainties, i.e., economic crises, immigration crises, unemployment, cultural change or terrorism, often spill over to focus attention on life, death, and existence, thus frequently fuel religion (e.g., Herriot, 2007). Indeed, researchers assert that religion helps its followers to understand and predict their environment, thus buffering them from the uncertainty associated with disruptions to order and meaning (Heine et al., 2006; Hogg et al., 2010; Inzlicht et al., 2011). This is possible because religious ideas tend to be highly stable, and indeed often evoke eternity. They explain the world as having a purpose, and they prescribe various kinds of values, moral or otherwise. The more simplistic, black–white, or dogmatic these beliefs are, the more order, predictability and certainty they provide (e.g., Altemeyer and Hunsberger, 1992; Brandt and Reyna, 2010). Thus, religious orthodoxy, characterized by a rigid, closed-minded, and dogmatic interpretation of religious contents (e.g., Wulff, 1991), may offer relief from uncertainty, by providing a sense of predictability and control (Inzlicht et al., 2009; Inzlicht and Tullett, 2010; Kossowska et al., 2016b; Sekerdej et al., in press).

Nevertheless, this process is not cost-free, as the simplified picture of the world that results is frequently distorted and has important behavioral consequences (e.g., Devine and Sharp, 2009; Smith et al., 2015). For example, one can easily imagine how the belief that one possesses the absolute truth can smoothly develop into negative attitudes toward those who do not accept it, let alone toward those who openly disagree with it. Indeed, many studies have demonstrated that religion is associated with more rather than less prejudice (e.g., Jackson and Hunsberger, 1999; Rowatt et al., 2005; Kossowska and Sekerdej, 2015; Sekerdej et al., in press). Recently, however, evidence has been found to suggest that religious people are selectively prejudiced (Batson et al., 1993; Rowatt et al., 2009), meaning that religious beliefs can encourage prejudice toward certain groups like homosexuals, single mothers, or atheists, but discourage prejudice toward other groups like the poor or older people (Kossowska and Sekerdej, 2015).

In the present study, we claim that religious orthodoxy is linked to prejudice, i.e., negative attitudes (Crandall et al., 2002), toward groups that in any way challenge religious worldviews or value systems. Atheists constitute the group that is supposedly the most threatening for orthodox believers. This is because atheists and religious people compete for the promotion of their own important moral values and belief systems (Jackson and Hunsberger, 1999). Religious people also tend to view atheists as distrustful, dissimilar, and to be violators of important norms and valued traditions (Beit-Hallahmi, 2010; Gervais, 2011; Gervais et al., 2011). The dissimilarity between these two groups itself may also cause threat, according to the idea that people seek to affirm the validity of their own values. In other words, in the face of groups perceived to be dissimilar to themselves, the sense of threat is awakened (e.g., Skitka et al., 2005; Brandt et al., 2014; Brandt and Van Tongeren, 2016; Sekerdej et al., in press).

Any kind of threat, such as that posed by value-violators, is an uncomfortable and aversive state, therefore, people generally feel the need to eliminate it (for an overview see Jonas et al., 2014). We claim that expressing prejudice may serve as an efficient strategy to reduce the threat posed by value-violators. This reasoning is consistent with research suggesting that prejudice and discrimination directed toward members of groups that violate important values, norms and traditions can be used to bolster one’s cultural worldview, and thus reduce threat (for an overview, see Burke et al., 2010). There is a body of research showing that threat increases group identification, adherence to group values, including religious ones (Hogg et al., 2010), and the propensity to defend them (McGregor et al., 2001; Hogg et al., 2007; for reviews see Kesebir and Pyszczynski, 2011; Jonas et al., 2014). In a similar vein, there is also experimental evidence showing that threat increases religious zeal (Hogg et al., 2010, for a review) that in turn can increase prejudice against those who do not share similar values. Our claim is also in line with studies showing that expressing prejudice against members of another group can buffer one’s self-esteem against self-threats (e.g., Fein and Spencer, 1997; Hogg, 2001; Collange et al., 2009). Thus, orthodox beliefs can lead people to reject or discriminate against those who might distort the neat and ordered picture of the world those beliefs provide; to push back against those who are thus perceived as violating important religious values.

Although a considerable amount of research has indeed demonstrated that the threat posed by value-violators leads to negative attitudes toward these groups (e.g., Hunsberger and Jackson, 2005; Brandt and Van Tongeren, 2016; Kossowska et al., 2016a; Sekerdej et al., in press), the hypothesis that expressing prejudice reduces threat has never been directly tested. Thus, we decided to test this hypothesis directly using cardiovascular reactivity, i.e., heart rate (HR), as a psychophysiological index of threat (Brehm et al., 1964; Croyle and Cooper, 1983; Elkin and Leippe, 1986; Losch and Cacioppo, 1990; Harmon-Jones et al., 1996; Robinson and Demaree, 2007; Butler et al., 2009). HR is a robust and “pure” measure of sympathetic and parasympathetic activity collected over short time intervals (Berntson et al., 1994; Cacioppo et al., 1994). Studies of stressor-induced HR reactivity have estimated its high internal consistency and test–retest reliability (Cacioppo, 1994; Uchino et al., 1995). Finally, HR plays a critical role in the regulation of both behavioral and physiological responses, including the regulation of “fear responses” (Milad et al., 2007; Delgado et al., 2008; Schiller et al., 2008). Increase in HR is also robustly elicited by social threat (Wager et al., 2009), something that makes it a useful measure for capturing perceptions of threat posed by value-violating groups.

Thus, we expect that people holding high (vs. low) levels of orthodox belief may respond with increased HR after being exposed to worldviews that challenge their value system. Moreover, we assume that highly orthodox participants will respond with decreased HR after having expressed prejudice toward the same outgroup, compared to those who will not have been given this opportunity. That is because expressing prejudice toward this particular group is a self-regulatory mechanism that helps highly orthodox people to reduce the feelings of threat posed by value-violating groups.

## Materials and Methods

### Participants

Before the experiment, a group of 581 volunteers (423 women, *M*_age_ = 28.51, *SD* = 82.17) were asked via an online questionnaire whether they believe in God (1 – yes, 2 – no) and if yes, they were asked to indicate their religious affiliation (Catholicism, Protestantism, Orthodoxy, Judaism, Islam, Hinduism, Buddhism, or other). Participants were recruited by an announcement via a local online social portal. They were also asked to complete the Right-Wing Authoritarianism Scale (Altemeyer, 1996; ratings were obtained on a 5-point scale, from 1 [*completely disagree*] to 5 [*completely agree*]; α = 0.68 *M* = 2.02; *SD* = 0.56) just to make the online study more believable for participants. We did not use the results of this scale or further analysis, as RWA was not of our interests.

Three hundred and forty-two people (270 women, *M*_age_ = 23.87, *SD* = 6.49) declared themselves to be believers, and from this group 266 (211 women, *M*_age_ = 24.19, *SD* = 12.35) described themselves as Catholics. This group was asked to complete the Post-Critical Belief scale (Duriez et al., 2000; 33 items). Ratings were obtained on a 7-point scale, from 1 (*completely disagree*) to 7 (*completely agree*). Based on their answers we calculated an orthodoxy index (α = 0.88, *M* = 3.21, *SD* = 1.43). Sample items of the orthodoxy scale are “I think that Bible stories should be taken literally, as they are written,” “God has been defined once and for all and is therefore immutable” and “Only the major religious traditions guarantee admittance to God.” This subscale was roughly normally distributed and was used to create two groups with higher (>90th percentile, *M* = 4.57; *SD* = 0.94) and lower (<10th percentile, *M* = 2.13; *SD* = 0.53) orthodoxy levels. The rationale for using groups extremely low and high in orthodoxy was the specificity of psychophysiological studies. Due to the fact that this type of study is difficult to run, it is a common practice to use small sample sizes. On the other hand, there is a risk that with small sample size the study would be underpowered. Therefore, we decided to increase the statistical power of the study by using a pre-selected sample of participants who were very high and very low in the crucial variable as suggested by Preacher et al. (2005). After screening, 38 participants were invited to the experiment (29 women, *M*_age_ = 24.32, *SD* = 7.02; in the highly orthodox group *N* = 17, 12 women, *M*_age_ = 23.94, *SD* = 6.18; in the low orthodox group *N* = 21, 17 women, *M*_age_ = 24.62, *SD* = 7.76).

An *a priori* power analysis (G^∗^Power 3.1.9.2, Faul et al., 2009) for large effect size of *f* = 0.50, revealed that a sample of 34 participants should be needed to reach statistical power at the recommended 0.80 level (Cohen, 1988) for the interaction effect. Thus, our sample size (*N* = 38) was sufficient to run all reported analyses. We assumed very large effect due to the non-probability sampling (only participants extreme in orthodoxy were invited for the study). This approach is commonly used to achieve greater statistical power in hypothesis testing (Preacher et al., 2005). Moreover, collecting the HR data in 256 Hz frequency should reduce statistical error and thus increase the effect size.

Participants were given a monetary compensation of 5 EUR for participation in the study. The study was carried out in accordance with the recommendations of the local Commission of Research Ethics, and included obtaining written informed consent from all subjects. An outlier analysis was carried out before the hypothesized model was tested. All measures, manipulations, and exclusions in the study are reported.

### Measures and Procedure

The study was carried out in a sound-attenuated room. After arriving at the lab, participants were asked to complete the Post-Critical Belief scale again in order to check the stability of the tool (α = 0.91, *M* = 3.10; *SD* = 1.59). Ratings were obtained on a 7-point scale, from 1 (*completely disagree*) to 7 (*completely agree*). Pre- and post-measures were strongly correlated, *r*(36) = 0.91, *p* < 0.001. Then, a baseline measurement of HR was taken for 2 min, during which participants were instructed to sit still and remain quiet. After that, sixteen stimulating slogans from an atheist march were displayed for 5,000 ms each on a computer screen (examples of slogans: *Secular Poland – Secular Europe; Sex Education not Parish Education; Free School – Religion back to Church*). The order of the slogans was randomized for each participant. Then, all 16 slogans were displayed once again at the same time and participants were asked to choose which one, in their opinion, is the most offensive for Catholics. We used this procedure to involve participants in careful reading of the slogans and thus strengthen our manipulation. Ninety percent of participants indicated the sentence, “*Every thinking man is an atheist*,” as the most offensive for Catholics.

Further, an experimental HR measurement was taken for 2 min and then participants were randomly assigned to one of two conditions. Low and high orthodox individuals were equally distributed to the control and the experimental condition. In the control group, participants were asked to fill out an openness questionnaire from the Five Factor Model (Costa and McCrae, 1992); in the experimental group they filled out the Negative Attitudes Toward Atheists scale, which was developed on the base of the measurement by Gervais (2011) (α = 0.93, *M* = 4.67, *SD* = 1.07), and included such items as, “I would be uncomfortable for me if an atheist taught my child” or “Atheists are a threat to the social order.” Participants assessed the items on a 9-point scale, from 1-totally disagree to 9-totally agree. The completion of the questionnaires in both groups lasted a comparable period of time.

**FIGURE 1:**
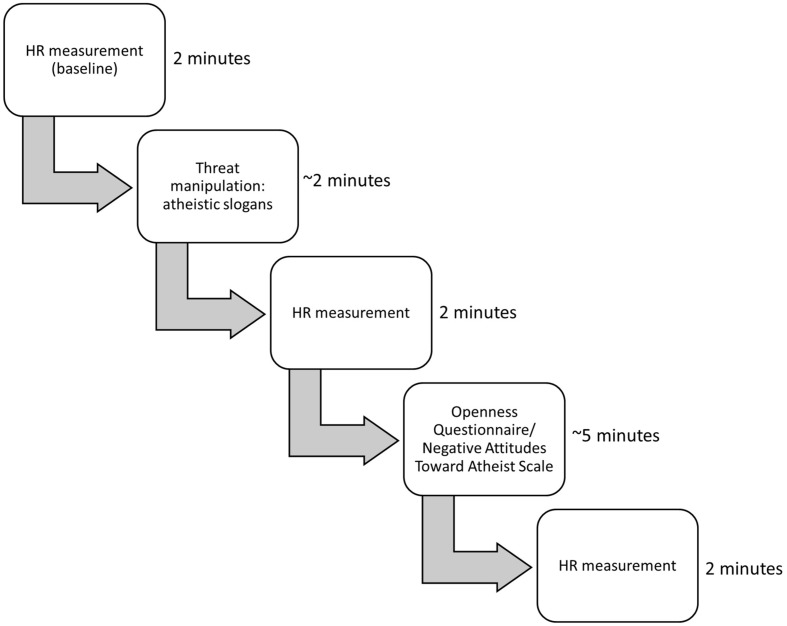
Outline of the study.

After completing the questionnaires, the second HR measurement was taken for 2 min. At the end, participants were thanked and debriefed. The outline of the study is presented on **Figure 1**. All computer tasks were displayed on a 19-inch LCD monitor. All experimental procedures were programmed in PsychoPy v. 1.82.01.

### Cardiovascular Data Pre-processing and Apparatus

In the presented study, threat was operationalized as HR level (Brehm et al., 1964). The electrocardiography (ECG) utilized the BioSemi ActiveTwo system with one electrode located under the left collarbone, acquired at a sampling rate of 256 Hz. Harvested data were off-line filtered using EDFbrowser software. Then, HR was separated from the ECG signal (Christov, 2004) for baseline and both first and second experimental measurements. To calculate HR as a dependent variable, baseline scores were subtracted from scores derived in each measurement (Llabre et al., 1991). As a result, we obtained two threat indices: HR1 and HR2 as indicators of threat after the threat manipulation, i.e., after reading atheistic slogans and after filling out the questionnaires, respectively.

## Results

Descriptive statistics for HR measurements are presented in **Table 1**. First, we examined if the baseline measurements of HR were reliable, i.e., if there were no differences between group in HR at the beginning of the experiment. To compare baseline HR for high and low orthodoxy group, we ran an independent-samples *t*-test, which confirmed that there was no significant difference between groups *t*(36) = -0.74, *p* = 0.465, 95% CI [-15.04, 7.01].

**Table 1:** Means, standard deviations (in parentheses) and change from baseline (DV) for HR taken during experiment for low and high orthodox individuals.

	Baseline		After manipulation		After control condition		After experimental condition	
				
Orthodoxy level	Low	High	Low	High	Low	High	Low	High
HR [bpm]	82.59 (15.95)	78.58 (17.50)	80.75 (10.61)	83.51 (21.27)	83.78 (14.54)	79.19 (29.24)	77.26 (10.80)	78.78 (14.09)
HR-baseline [dv]			-1.84 (9.22)	4.93 (7.15)	-2.94 (5.59)	1.00 (7.18)	1.61 (7.31)	-4.01 (4.35)


To check the dynamics of HR during the whole study we calculated HR index: HR2-HR1. Positive value means that there was increase in HR after manipulation, while negative index means that there was decrease in HR. We conducted this analysis with this new index as dependent variable, controlling for baseline level of HR. Main effects of group (orthodoxy) and condition were insignificant. The interaction between orthodoxy and condition was significant *F*(1,33) = 7.25, *p* = 0.011, $\eta_{p}^{2}$ = 0.18. We ran Bonferroni corrected pairwise comparisons, which showed differences between experimental and control conditions only for high orthodox participants, *F*(1,33) = 11.48, *p* = 0.002, $\eta_{p}^{2}$ = 0.26. In experimental condition the decrease in HR for high orthodox participants was significantly greater than in control condition (*M* = -7.71 bpm; *SD* = 4.35; *M* = -0.34 bpm; *SD* = 7.18, respectively). For low orthodox participants there were no difference between the conditions *F*(1,33) = 0.37, *p* = 0.545, $\eta_{p}^{2}$ = 0.01.

Although the above analysis allowed to studying the dynamic of HR in both, high and low orthodox groups, they are also problematic. It is because during HR1 measurement participants were not yet assigned to any of the conditions. Thus, to make the picture clear we decided to treat HR2 as a dependent measure with controlling HR1 since we are interested mainly in interaction between orthodoxy and the conditions. We checked if there was a difference in HR after the threat manipulation, i.e., facing atheistic slogans, between the high and low orthodox groups. An independent-samples *t*-test was conducted to compare HR1 for high and low orthodoxy group. According to our assumptions, participants high in orthodoxy obtained significantly higher levels of HR (*M* = 4.93 bpm; *SD* = 7.15) than those low in orthodoxy (*M* = -1.84 bpm; *SD* = 9.22); *t*(36) = 2.48, *p* = 0.018, 95% CI [1.24, 12.30].

In the next step, we checked if participants high and low in orthodoxy differed on HR levels across conditions (experimental vs. control). Thus, we ran an ANOVA with high vs. low orthodox groups and conditions (experimental vs. control) as between-subject factors and HR2 as the dependent variable. In order to take into account input levels of arousal, we controlled for HR1. The main effect of group was *F*(1,33) = 0.26, *p* = 0.617, $\eta_{p}^{2}$ = 0.008 and the effect of condition was *F*(1,33) = 0.02, *p* = 0.885, $\eta_{p}^{2}$ = 0.001. As we predicted, the two-way interaction turned out to be significant *F*(1,33) = 9.63, *p* = 0.004, $\eta_{p}^{2}$ = 0.226.

**FIGURE 2:**
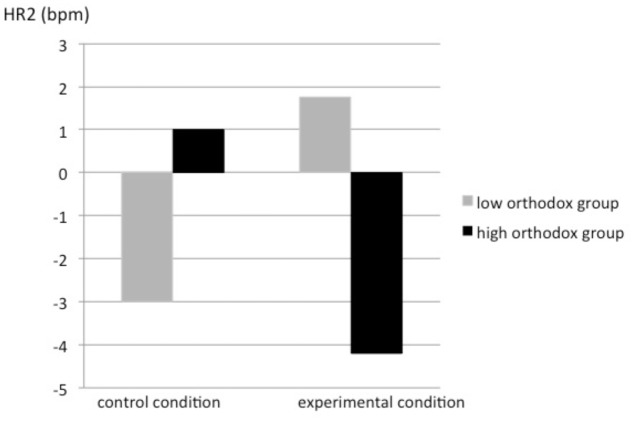
Threat measured by HR as a function of levels of religious orthodoxy and condition (experimental vs. control). HR2 was calculated by subtracting the baseline HR measurement from the second HR measurement.

For the two-way interaction (**Figure 2**), Bonferroni corrected pairwise comparisons showed differences between the experimental and control conditions for highly orthodox participants, *F*(1,33) = 4.71, *p* = 0.037, $\eta_{p}^{2}$ = 0.125. Participants high in orthodoxy had lower and significantly reduced HR in the experimental condition (*M* = -4.01 bpm; *SD* = 4.35), than in the control condition (*M* = 1.00 bpm; *SD* = 7.18). However, for low orthodoxy participants there was also a difference between the conditions, but of marginal significance, *F*(1,33) = 4.08, *p* = 0.051, $\eta_{p}^{2}$ = 0.110. In the experimental condition, they had higher HR (*M* = 1.61 bpm; *SD* = 7.31) than in the control condition (*M* = -2.94 bpm; *SD* = 5.59).

We also tested whether highly orthodox participants indeed expressed prejudice after the threat manipulation. As we predicted, highly orthodox participants revealed higher levels of prejudice against atheists (*M* = 5.69; *SD* = 1.84), than those with low orthodoxy level (*M* = 2.90; *SD* = 1.39). Comparisons between groups were significant, *t*(17) = 3.75, *p* = 0.002, 95% CI [1.22, 4.36].

## Discussion

In the present study we observed that people who hold high (vs. low) levels of orthodox beliefs are more sensitive to the threat manipulation, i.e., after being exposed to atheistic slogans participants who were high in orthodox belief, but not those who were low in orthodox belief, responded with increased HR. This result provides empirical support for the claim that religion, especially in its orthodox form, although offering relief from distress and uncertainty (e.g., Inzlicht and Tullett, 2010), at the same time makes believers especially sensitive to violations of those religious beliefs. If orthodox beliefs act as a shield against uncertainty, any violations of such system are regarded as highly threatening. This is in line with previous studies suggesting that religious people respond with increased threat to any group that promotes alternative values or beliefs systems (see: Rowatt et al., 2014; Kossowska et al., 2016a).

Moreover, our results suggest that prejudice toward a value-violating group serves as an efficient strategy for reducing feelings of threat posed by that group. We observed that highly orthodox people responded with decreased HR after expressing prejudice against atheists, but with increased HR when they had no possibility to behave in a prejudiced way. Thus, expressing prejudice seems to be a self-regulatory mechanism, as self-regulation is the process by which a system uses information about its present state to change that state in the pursuit of goals (McCullough and Boker, 2007). It seems that when religious worldviews that are essential for orthodox believers are compromised by the worldviews of other groups, they have the tendency to reject these groups and this rejection seems to be an adaptive strategy that helps to reduce threat. This is an important finding as previous studies have also claimed that prejudice is a reaction to threat and that it may play a self-regulatory functions, without providing the kind of direct empirical support we report here (e.g., Reyna et al., 2006, 2009; Henry and Reyna, 2007; Wetherell et al., 2013).

We suggest that expressing prejudice helps to cope with the threat posed by value-violators, and that the validity of a cultural worldview and the standards and values associated with that worldview buffers against threat. Thus, expressing prejudice may reduce threat because it bolsters one’s cultural worldview (e.g., Kesebir and Pyszczynski, 2011). It is also possible that expressing prejudice against members of a threatening group can buffer one’s self-esteem against self-threats (e.g., Abrams and Hogg, 1990). However, our study did not allow us to identify the exact mechanism of threat reduction. In addition, it is worth noticing that the measure of prejudice toward atheists we used in the study might be understood not only as expressions of prejudice, but also s affirmations of one’s worldview. This issue calls for another study.

Although in this study we were interested in the response patterns of highly orthodox people, the way that people low in orthodoxy responded in our procedure is also informative. First, it is worth noticing that being confronted with atheistic slogans, low orthodox individuals responded with decreased HR. It seems that these slogans do not pose a threat to their worldviews and that they feel comfortable with such content. Second, they responded with increased HR while completing the prejudice questionnaire. This result suggests that they felt uncomfortable, as they were asked to behave in a way that was not elicited by their religious worldview. People expressing low levels of religious orthodoxy are more willing to respect other people’s choice to be religious or non-religious, and they try to find a personally meaningful interpretation of their beliefs that can be either religious or non-religious (e.g., Wulff, 1991).

The question arises, however, whether prejudice is the only self-regulatory strategy used to reduce the threat posed by value-violating groups. Studies have demonstrated that people can reduce threat in many different ways, e.g., by pursuing concrete goals (e.g., chocolate, gambling for money) or abstract goals such as ideals, ideologies, and beliefs (for an overview see Jonas et al., 2014). The most effective strategies, however, are social strategies, meaning those commitments that are nested within social contexts, that involve social support in interpersonal relations, or that rely on social identities in group-related contexts (Jonas et al., 2014). Therefore, despite rejecting an outgroup, one may rely on social strategies that promote support for the ingroup, e.g., by showing increased pro-social attitudes, such as the donation of money to ingroup charities (Jonas et al., 2002), collective action intentions (Fritsche et al., 2013), or promoting ingroup symbols and products (e.g., Germans favoring German restaurants, cars, talk shows, cities, or money; Jonas et al., 2005). Prejudice does, however, fit well with these other lines of research, as it is a way of consolidating the ingroup by pitting it against an outgroup(s). As we did not study ingroup attitudes, the question whether ingroup favoritism is an equally effective strategy to reduce threat remains open.

We claim that the effect we found is probably not unique to religion *per se*, and that it would occur with any form of ideological commitment. There is some research showing that in the absence of religious beliefs, secular beliefs based on science or reason can replace religion as a source of comfort and meaning (e.g., Wulff, 1991; Arndt et al., 2002; Proulx, 2009; Farias et al., 2013). Thus, we would expect that expressing prejudice toward value-violators is an adaptive strategy for any kind of strong and dogmatic believers for whom competition surrounding relevant value promotion is important (Kossowska et al., 2016a). On the other hand, as highly orthodox people’s dominant motivational concern is the preservation of safety and security (Inzlicht and Tullett, 2010), threat-relevant information should be seen as especially informative and valuable. In addition, they may overestimate the threat. Thus, they may react to generalized threat by defending their attitudes, values, and worldviews with intolerance toward people with differing beliefs, biased processing of attitude-inconsistent information, and the affirmation of core values. This issue also calls for further research.

Finally, we would like to highlight some limitations of our study. First, we presented only one study; a study that was conducted on a relatively small, preselected, and very homogeneous sample composed only of Catholics. Hence, the generalizability of our results is still an open question and requires further research. Likewise, in the present research we focused on broad categories of religiosity, such as religious orthodoxy, and as the outgroup we used only atheists. Therefore, in future research various finer-grained aspects of religious beliefs can be explored, as well as different outgroups. Finally, it is worth noting that although HR is a reliable index of threat (Brehm et al., 1964), some have argued HR to be an index of non-specific arousal (Azarbarzin et al., 2014). However, due to the biological reasons for tachycardia (the HR increasing in the resting state) we believe that HR is mostly linked to threat, anxiety and stress. The large body of medical and biological research supports this assumption (for example: Brodsky et al., 1987; Tornatzky and Miczek, 1994). However, in follow-up studies it would nevertheless be useful to include additional measures of threat apart from the HR index. This would allow for the further study of how the expression of prejudice may indeed reduce the perception of threat from value-violating outgroups.

## Ethics Statement

This study was carried out in accordance with the recommendations of in ethical guidelines of The Code of Ethics of the World Medical Association (Declaration of Helsinki). All subjects gave written informed consent in accordance with the Declaration of Helsinki. The protocol was approved by Commission of Research Ethics, the Institute of Psychology, Jagiellonian University.

## Author Contributions

MK developed the rationale for the study and wrote the manuscript. AC-K, PS, MS, and MK contributed to the study concept and study design. PS contributed to data collection and with MW performed the data analyses. All authors approved the final version of the manuscript for submission.

## Conflict of Interest Statement

The authors declare that the research was conducted in the absence of any commercial or financial relationships that could be construed as a potential conflict of interest.

## References

[B1] Abrams D. Hogg M. A. (1990). Social identification, self-categorization and social influence. *Eur. Rev. Soc. Psychol.* 1 195–228. 10.1080/14792779108401862

[B2] Altemeyer B. (1996). *The Authoritarian specter.* London: Cambridge and Harvard University Press.

[B3] Altemeyer B. Hunsberger B. E. (1992). Authoritarianism, religious fundamentalism, quest, and prejudice. *Int. J. Psychol. Relig.* 2 113–133. 10.1207/s15327582ijpr0202_5

[B4] Arndt J. Greenberg J. Cook A. (2002). Mortality salience and the spreading activation of worldview-relevant constructs: exploring the cognitive architecture of terror management. *J. Exp. Psychol. Gen.* 131 307–324. 10.1037/0096-3445.131.3.307 12214749

[B5] Azarbarzin A. Ostrowski M. Hanly P. Younes M. (2014). Relationship between arousal intensity and heart rate response to arousal. *Sleep* 37 645–653. 10.5665/sleep.3560 24899756 PMC4044744

[B6] Batson C. D. Schoenrade P. Ventis W. L. (1993). *Religion and the Individual: A Social-Psychological Perspective.* New York, NY: Oxford University Press.

[B7] Beit-Hallahmi B. (2010). “Morality and immorality among the irreligious,” in *Atheism and Secularity*, ed. Zuckerman P. (Westport, CT: Greenwood), 113–148.

[B8] Berntson G. G. Cacioppo J. T. Binkley P. F. Uchino B. N. Quigley K. S. Fieldstone A. (1994). Autonomic cardiac control. III. Psychological stress and cardiac response in autonomic space as revealed by pharmacological blockades. *Psychophysiology* 31 599–608. 10.1111/j.1469-8986.1994.tb02352.x 7846220

[B9] Brandt M. J. Reyna C. (2010). The role of prejudice and the need for closure in religious fundamentalism. *Pers. Soc. Psychol. Bull.* 36 715–725. 10.1177/0146167210366306 20348439

[B10] Brandt M. J. Reyna C. Chambers J. R. Crawford J. T. Wetherell G. (2014). The ideological-conflict hypothesis: intolerance among both liberals and conservatives. *Curr. Dir. Psychol. Sci.* 23 27–34. 10.1177/0963721413510932

[B11] Brandt M. J. Van Tongeren D. R. (2016). People both high and low on religious fundamentalism are prejudiced toward dissimilar groups. *J. Pers. Soc. Psychol.* 112 76–97. 10.1037/pspp0000076 26523998

[B12] Brehm M. L. Back K. W. Bogdonoff M. D. (1964). A physiological effect of cognitive dissonance under stress and deprivation. *J. Abnorm. Soc. Psychol.* 69 303–310. 10.1037/h0041671 14218284

[B13] Brodsky M. A. Sato D. A. Iseri L. T. Wolff L. J. Allen B. J. (1987). Ventricular tachyarrhythmia associated with psychological stress. The role of the sympathetic nervous system. *JAMA* 257 2064–2067. 10.1001/jama.1987.03390150080039 2882033

[B14] Burke B. L. Martens A. Faucher E. H. (2010). Two decades of terror management theory: a meta-analysis of mortality salience research. *Pers. Soc. Psychol. Rev.* 14 155–195. 10.1177/1088868309352321 20097885

[B15] Butler E. A. Lee T. L. Gross J. J. (2009). Does expressing your emotions raise or lower your blood pressure? The answer depends on cultural context. *J. Cross Cult. Psychol.* 40 510–517. 10.1177/0022022109332845 25505801 PMC4260334

[B16] Cacioppo J. T. (1994). Social neuroscience: autonomic, neuroendocrine, and immune responses to stress. *Psychophysiology* 31 113–128. 10.1111/j.1469-8986.1994.tb01032.x 8153248

[B17] Cacioppo J. T. Berntson G. G. Binkley P. F. Quigley K. S. Uchino B. N. Fieldstone A. (1994). Autonomic cardiac control. II. Noninvasive indices and basal response as revealed by autonomic blockades. *Psychophysiology* 31 586–598. 10.1111/j.1469-8986.1994.tb02351.x 7846219

[B18] Christov I. I. (2004). Real time electrocardiogram QRS detection using combined adaptive threshold. *Biomed. Eng. Online* 3:28 10.1186/1475-925X-3-28 PMC516783 15333132

[B19] Cohen J. (1988). *Statistical Power Analysis for the Behavioral Sciences*, 2nd Edn Hillsdale, NJ: Erlbaum.

[B20] Collange J. Fiske S. Santioso R. (2009). Maintaining a positive self-image by stereotyping others: self-threat and the stereotype content model. *Soc. Cogn.* 27 138–149. 10.1521/soco.2009.27.1.138 PMC3882017 24403668

[B21] Costa P. T. McCrae R. R. (1992). *Revised NEO Personality Inventory (NEO-PI-R) and NEO Five Factor Inventory (NEO- FFI): Professional Manual.* Odessa, FL: Psychological Assessment Resources.

[B22] Crandall C. S. Eshleman A. O’brien L. (2002). Social norms and the expression and suppression of prejudice: the struggle for internalization. *J. Pers. Soc. Psychol.* 82 359–378. 10.1037/0022-3514.82.3.359 11902622

[B23] Croyle R. T. Cooper J. (1983). Dissonance arousal: physiological evidence. *J. Pers. Soc. Psychol.* 45 782–791. 10.1037/0022-3514.45.4.782 6631664

[B24] Delgado M. R. Gillis M. M. Phelps E. A. (2008). Regulating the expectation of reward via cognitive strategies. *Nat. Neurosci.* 11 880–881. 10.1038/nn.2141 18587392 PMC3077128

[B25] Devine P. G. Sharp L. B. (2009). “Automaticity and Control in Stereotyping and Prejudice,” in *Handbook of Prejudice, Stereotyping, and Discrimination*, ed. Nelson T. D. (New York, NY: Psychology Press), 61–87.

[B26] Duriez B. Fontaine J. R. J. Hutsebaut D. (2000). A further elaboration of the Post-Critical Belief scale: evidence for the existence of four different approaches to religion in Flanders-Belgium. *Psychol. Belg.* 40 153–181.

[B27] Elkin R. A. Leippe M. R. (1986). Physiological arousal, dissonance, and attitude change: evidence for a dissonance-arousal link and a “don’t remind me” effect. *J. Pers. Soc. Psychol.* 51 55–65. 10.1037/0022-3514.51.1.55 3735071

[B28] Farias M. Newheiser A. K. Kahane G. de Toledo Z. (2013). Scientific faith: belief in science increases in the face of stress and existential anxiety. *J. Exp. Soc. Psychol.* 49 1210–1213. 10.1016/j.jesp.2013.05.008 24187384 PMC3807800

[B29] Faul F. Erdfelder E. Buchner A. Lang A. G. (2009). Statistical power analyses using G^∗^ Power 3.1: tests for correlation and regression analyses. *Behav. Res. Methods* 41 1149–1160. 10.3758/BRM.41.4.1149 19897823

[B30] Fein S. Spencer S. J. (1997). Prejudice as self-image maintenance: affirming the self through derogating others. *J. Pers. Soc. Psychol.* 73 31–44. 10.1037/0022-3514.73.1.31

[B31] Fritsche I. Jonas E. Ablasser C. Beyer M. Kuban J. Manger A. M. (2013). The power of we: evidence for group-based control. *J. Exp. Soc. Psychol.* 49 19–32. 10.1016/j.jesp.2012.07.014

[B32] Gervais W. M. (2011). Finding the faithless: perceived atheist prevalence reduces anti-atheist prejudice. *Pers. Soc. Psychol. Bull.* 37 543–556. 10.1177/0146167211399583 21343437

[B33] Gervais W. M. Shariff A. F. Norenzayan A. (2011). Do you believe in atheists? Distrust is central to anti-atheist prejudice. *J. Pers. Soc. Psychol.* 101 1189–1206. 10.1037/a0025882 22059841

[B34] Harmon-Jones E. Greenberg J. Solomon S. Simon L. (1996). The effects of mortality salience on intergroup bias between minimal groups. *Eur. J. Soc. Psychol.* 26 677–681. 10.1002/(SICI)1099-0992(199607)26:4<677::AID-EJSP777<3.0.CO;2-2

[B35] Heine S. J. Proulx T. Vohs K. D. (2006). The meaning maintenance model: on the coherence of social motivations. *Pers. Soc. Psychol. Rev.* 10 88–110. 10.1207/s15327957pspr1002_1 16768649

[B36] Henry P. J. Reyna C. (2007). Value judgments: the impact of perceived value violations on political attitudes. *Polit. Psychol.* 28 273–298. 10.1111/j.1467-9221.2007.00569.x

[B37] Herriot P. (2007). *Religious Fundamentalism and Social Identity.* New York, NY: Routledge.

[B38] Hogg M. A. (2001). “Self-categorization and subjective uncertainty resolution: cognitive and motivational and facets of social identity and group membership,” in *The Social Mind: Cognitive and Motivational Aspects of Interpersonal Behaviour*, eds Forgas J. P. Williams K. D. Wheeler L. (New York, NY: Cambridge University Press), 323–349.

[B39] Hogg M. A. Adelman J. R. Blagg R. D. (2010). Religion in the face of uncertainty: an uncertainty-identity theory account of religiousness. *Pers. Soc. Psychol. Rev.* 14 72–83. 10.1177/1088868309349692 19855094

[B40] Hogg M. A. Sherman D. K. Dierselhuis J. Maitner A. T. Moffitt G. (2007). Uncertainty, entitativity, and group identification. *J. Exp. Soc. Psychol.* 43 135–142. 10.1016/j.jesp.2005.12.008

[B41] Hunsberger B. Jackson L. M. (2005). Religion, meaning, and prejudice. *J. Soc. Issues* 61 807–826. 10.1111/j.1540-4560.2005.00433.x

[B42] Inzlicht M. McGregor I. Hirsh J. B. Nash K. (2009). Neural markers of religious conviction. *Psychol. Sci.* 20 385–392. 10.1111/j.1467-9280.2009.02305.x 19291205

[B43] Inzlicht M. Tullett A. M. (2010). Reflecting on God: religious primes can reduce neurophysiological response to errors. *Psychol. Sci.* 21 1184–1190. 10.1177/0956797610375451 20558751

[B44] Inzlicht M. Tullett A. M. Good M. (2011). The need to believe: a neuroscience account of religion as a motivated process. *Religion Brain Behav.* 1 192–212. 10.1080/2153599X.2011.647849

[B45] Jackson L. M. Hunsberger B. (1999). An intergroup perspective on religion and prejudice. *J. Sci. Study Relig.* 38 509–523. 10.2307/1387609

[B46] Jonas E. Fritsche I. Greenberg J. (2005). Currencies as cultural symbols–an existential psychological perspective on reactions of Germans toward the Euro. *J. Econ. Psychol.* 26 129–146. 10.1016/j.joep.2004.02.003

[B47] Jonas E. McGregor I. Klackl J. Agroskin D. Fritsche I. Holbrook C. (2014). Threat and defense: from anxiety to approach. *Adv. Exp. Soc. Psychol.* 49 219–286. 10.1016/B978-0-12-800052-6.00004-4

[B48] Jonas E. Schimel J. Greenberg J. Pyszczynski T. (2002). The scrooge effect: evidence that mortality salience increases prosocial attitudes and behavior. *Pers. Soc. Psychol. Bull.* 28 1342–1353. 10.1177/014616702236834

[B49] Kesebir P. Pyszczynski T. (2011). A moral-existential account of the psychological factors fostering intergroup conflict. *Soc. Personal. Psychol. Compass* 5 878–890. 10.1111/j.1751-9004.2011.00397.x

[B50] Kossowska M. Czernatowicz-Kukuczka A. Sekerdej M. (2016a). Many faces of dogmatism: prejudice as a way of protecting certainty against value violators among dogmatic believers and atheists. *Br. J. Psychol.* 108 127–147. 10.1111/bjop.12186 26892769

[B51] Kossowska M. Sekerdej M. (2015). Searching for certainty: religious beliefs and intolerance toward value-violating groups. *Pers. Individ. Dif.* 83 72–76. 10.1016/j.paid.2015.03.053

[B52] Kossowska M. Szwed P. Wronka E. Czarnek G. Wyczesany M. (2016b). Anxiolytic function of fundamentalist beliefs: neurocognitive evidence. *Pers. Individ. Dif.* 101 390–395. 10.1016/j.paid.2016.06.039

[B53] Llabre M. M. Spitzer S. B. Saab P. G. Ironson G. H. Schneiderman N. (1991). The reliability and specificity of delta versus residualized change as measures of cardiovascular reactivity to behavioral challenges. *Psychophysiology* 28 701–711. 10.1111/j.1469-8986.1991.tb01017.x 1816598

[B54] Losch M. Cacioppo J. (1990). Cognitive dissonance may enhance sympathetic tonus, but attitudes are changed to reduce negative affect rather than arousal. *J. Exp. Soc. Psychol.* 26 289–304. 10.1016/0022-1031(90)90040-S

[B55] McCullough M. E. Boker S. M. (2007). “Dynamical modeling for studying self-regulatory processes: an example from the study of religious development over the life span,” in *Handbook of Methods in Positive Psychology*, eds Ong A. D. Dulmen M. V. (New York, NY: Oxford University Press), 380–394.

[B56] McGregor I. Zanna M. P. Holmes J. G. Spencer S. J. (2001). Compensatory conviction in the face of personal uncertainty: going to extremes and being oneself. *J. Pers. Soc. Psychol.* 80 472–488. 10.1037/0022-3514.80.3.472 11300580

[B57] Milad M. Wright C. Orr S. Pitman R. Quirk G. Rauch S. (2007). Recall of fear extinction in humans activates the ventromedial prefrontal cortex and hippocampus in concert. *Biol. Psychiatry* 62 1191–1194. 10.1016/j.biopsych.2006.10.011 17217927

[B58] Preacher K. J. Rucker D. D. MacCallum R. C. Nicewander W. A. (2005). Use of the extreme groups approach: a critical reexamination and new recommendations. *Psychol. Methods* 10 178–192. 10.1037/1082-989X.10.2.178 15998176

[B59] Proulx T. (2009). The Feeling of the absurd: towards an integrative theory of sense-making. *Psychol. Inq.* 20 230–234. 10.1080/10478400903333494

[B60] Reyna C. Brandt M. Viki T. (2009). Blame it on hip-hop: anti-rap attitudes as a proxy for prejudice. *Group Process. Intergroup Relat.* 12 361–380. 10.1177/1368430209102848

[B61] Reyna C. Henry P. J. Korfmacher W. Tucker A. (2006). Attributional stereotypes as cues for deservingness: examining the role of principled conservatism in racial policy. *J. Pers. Soc. Psychol.* 90 109–128. 10.1037/0022-3514.90.1.109 16448313

[B62] Robinson J. L. Demaree H. A. (2007). Physiological and cognitive effects of expressive dissonance. *Brain Cogn.* 63 70–78. 10.1016/j.bandc.2006.08.003 17046129

[B63] Rowatt W. C. Carpenter T. Haggard M. (2014). “Religion, prejudice, and intergroup relations,” in *Religion, Personality, and Social Behavior*, ed. Saroglou V. (New York, NY: Psychology Press), 170–192.

[B64] Rowatt W. C. Franklin L. M. Cotton M. (2005). Patterns and personality correlates of implicit and explicit attitudes toward Christians and Muslims. *J. Sci. Study Relig.* 44 29–43. 10.1111/j.1468-5906.2005.00263.x

[B65] Rowatt W. C. LaBouff J. Johnson M. Froese P. Tsang J.-A. (2009). Associations among religiousness, social attitudes, and prejudice in a national random sample of American adults. *Psychol. Relig. Spiritual.* 1 14–24. 10.1037/a0014989

[B66] Schiller D. Levy I. Niv Y. Ledoux J. E. Phelps E. A. (2008). From fear to safety and back: reversal of fear in the human brain. *J. Neurosci.* 28 11517–11525. 10.1523/JNEUROSCI.2265-08.2008 18987188 PMC3844784

[B67] Sekerdej M. Kossowska M. Czernatowicz-Kukuczka A. (in press) Uncertainty and prejudice: the role of religiosity in shaping group attitudes. *Eur. J. Soc. Psychol.*

[B68] Skitka L. J. Bauman C. W. Sargis E. G. (2005). Moral conviction: another contributor to attitude strength or something more? *J. Pers. Soc. Psychol.* 88 895–917. 10.1037/0022-3514.88.6.895 15982112

[B69] Smith E. R. Mackie D. M. Claypool H. M. (2015). *Social Psychology*, 4th Edn New York, NY: Psychology Press.

[B70] Tornatzky W. Miczek K. A. (1994). Behavioral and autonomic responses to intermittent social stress: differential protection by clonidine and metoprolol. *Psychopharmacology* 116 346–356. 10.1007/BF02245339 7892426

[B71] Uchino B. N. Cacioppo J. T. Malarkey W. Glaser R. (1995). Individual differences in cardiac sympathetic control predict endocrine and immune responses to acute psychological stress. *J. Pers. Soc. Psychol.* 69 736–743. 10.1037/0022-3514.69.4.736 7473028

[B72] Wager T. D. Waugh C. E. Lindquist M. Noll D. C. Fredrickson B. L. Taylor S. F. (2009). Brain mediators of cardiovascular responses to social threat: part I: reciprocal dorsal and ventral sub-regions of the medial prefrontal cortex and heart-rate reactivity. *Neuroimage* 47 821–835. 10.1016/j.neuroimage.2009.05.043 19465137 PMC3275821

[B73] Wetherell G. Brandt M. J. Reyna C. (2013). Discrimination across the ideological divide: the role of value violations and abstract values in discrimination by liberals and conservatives. *Soc. Psychol. Pers. Sci.* 4 658–667. 10.1177/1948550613476096

[B74] Wulff D. M. (1991). *Psychology of Religion: Classic and Contemporary Views.* New York, NY: Wiley.

